# Scoping out noisy galvanic vestibular stimulation: a review of the parameters used to improve postural control

**DOI:** 10.3389/fnins.2023.1156796

**Published:** 2023-05-02

**Authors:** Ruth McLaren, Paul F. Smith, Rachael L. Taylor, Imran Khan Niazi, Denise Taylor

**Affiliations:** ^1^Rehabilitation Innovation Centre, Health and Rehabilitation Research Institute, School of Clinical Sciences, Auckland University of Technology, Auckland, New Zealand; ^2^Eisdell Moore Centre for Hearing and Balance Research, University of Auckland, Auckland, New Zealand; ^3^Department of Pharmacology and Toxicology, School of Biomedical Sciences, The Brain Health Research Centre, University of Otago, Dunedin, New Zealand; ^4^Department of Physiology, University of Auckland, Auckland, New Zealand; ^5^Centre of Chiropractic Research, New Zealand College of Chiropractic, Auckland, New Zealand; ^6^Centre for Sensory-Motor Interactions, Department of Health Science and Technology, Aalborg University, Aalborg, Denmark

**Keywords:** neuromodulation, nGVS, posture, galvanic vestibular stimulation, balance, stochastic resonance, vestibular, parameters

## Abstract

**Objective:**

Noisy galvanic vestibular stimulation (nGVS) has been used to facilitate vestibular function and improve gait and balance in people with poor postural control. The aim of this scoping review is to collate, summarize and report on the nGVS parameters that have been used to augment postural control.

**Method:**

A systematic scoping review was conducted up to December 2022. Data were extracted and synthesized from 31 eligible studies. Key nGVS parameters were identified, and the importance of these parameters and their influence on postural control evaluated.

**Results:**

A range of nGVS parameters have been used to augment postural control, including; noise waveform, amplitude, frequency band, duration of stimulation, method of amplitude optimization, size and composition of electrodes and the electrode skin interface.

**Conclusion:**

Systematic evaluation of the individual parameters that can be manipulated in the nGVS waveform identified that a broad array of settings have been utilized in each parameter across the studies. Choices made around the electrode and electrode-skin interface, as well as the amplitude, frequency band, duration and timing of the waveform are likely to influence the efficacy of nGVS. The ability to draw robust conclusions about the selection of optimal nGVS parameters to improve postural control, is hindered by a lack of studies that directly compare parameter settings or consider the variability in individuals’ response to nGVS. We propose a guideline for the accurate reporting of nGVS parameters, as a first step toward establishing standardized stimulation protocols.

## Introduction

Loss of postural control is a common consequence of both normal aging and pathological processes such as vestibular or other neurological disease (Agrawal et al., 2020a). Postural control requires appropriate and timely motor responses and is dependent on inputs from multiple sensory systems (vestibular, visual, and proprioceptive), integrated in the central nervous system (Agrawal et al., 2020b). In situations where a loss of postural control is attributed to a failure of vestibular information, there is an increased dependance on visual and proprioceptive information for balance (Sprenger et al., 2017). This can be inadequate in situations where visual and proprioceptive information is less available, or less accurate (Sprenger et al., 2017).

Noisy galvanic vestibular stimulation (nGVS), also known as stochastic vestibular stimulation (SVS), has been investigated as a treatment to improve postural control in standing and walking in healthy adults (Fujimoto et al., 2016; Wuehr et al., 2016b; Temple et al., 2018; Inukai et al., 2018a,b; Fujimoto et al., 2019; Inukai et al., 2020a,b,c; Nooristani et al., 2021), and in people with bilateral vestibulopathy (Iwasaki et al., 2014; Wuehr et al., 2016a; Fujimoto et al., 2018; Iwasaki et al., 2018; Ko et al., 2020; Sprenger et al., 2020; Chen et al., 2021; Eder et al., 2022), Parkinson’s disease (Pal et al., 2009; Samoudi et al., 2015; Wuehr et al., 2022) and multiple sclerosis (Lotfi et al., 2021). nGVS is a zero mean noisy galvanic current applied to the vestibular apparatus and its afferent nerves *via* electrodes placed bilaterally over the mastoid processes (Lajoie et al., 2021). It has been demonstrated to be safe, with infrequent adverse effects that have been of a mild nature (Wilkinson et al., 2009; Utz et al., 2011; Dilda et al., 2012; Lotfi et al., 2021; Matsugi et al., 2021).

**Figure 1:**
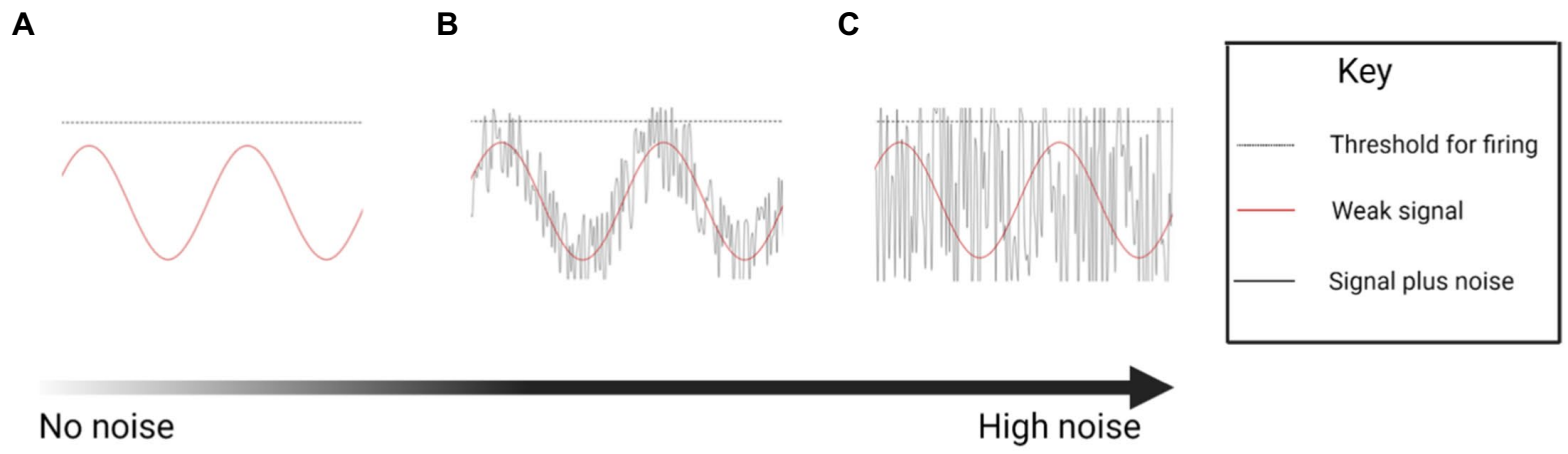
Conceptual drawing illustrating the theoretical stochastic effect of increasing levels of noise on firing of a neuron (Created with BioRender.com). **(A)** A sine wave in which the signal fails to reach the threshold for neural firing. **(B)** A sine wave enhanced by an optimum noise signal, boosting the signal past the threshold for firing. **(C)** Excessive noise boosts the sine wave indiscriminately, increasing neural firing, but losing the temporal information.

While the exact mechanism of nGVS facilitation of postural control is unknown, it is assumed that there is an element of sensory restoration *via* stochastic resonance. nGVS boosts the weak physiological signal from the vestibular apparatus, reducing the firing threshold of vestibular irregular afferent neurons (Figure 1) (Moss et al., 2004; Yu et al., 2012; Schniepp et al., 2018; Wuehr et al., 2018; Dlugaiczyk et al., 2019). However, this response is dependent on the parameters of the noisy signal. Optimal noise parameters will enhance the physiological signal, whereas too weak a noise signal will have no effect, and too high a noise signal will degrade the information content of the physiological signal (Figure 1)(Moss et al., 2004).

While recent reviews have confirmed the beneficial effect of nGVS in healthy people (Stefani et al., 2020), people with BVP (McLaren et al., 2022), and Parkinson’s disease (Mahmud et al., 2022), improvements have not been uniform. Sprenger et al. (2020), and Nooristani et al. (2019b) found no improvement in standing balance with nGVS compared to sham controls, and Matsugi et al. (2020) and Sprenger et al. (2020) even found nGVS increased sway while standing on foam.

To develop clinical applications for nGVS it is critical that we understand which nGVS parameters will consistently and sustainably improve postural control. To date, limited systematic evaluation of parameter settings, and the variability in individual’s response to nGVS, make it unclear how parameter choice influences responsiveness and outcomes. This has resulted in a lack of consensus on the specific nGVS parameter settings to optimize postural control; frequency band, amplitude, noise waveform, electrode size, shape and composition, optimisation procedure and duration of stimulation (Dlugaiczyk et al., 2019; Fujimoto et al., 2019; Herssens and McCrum, 2019; Stefani et al., 2020; Lajoie et al., 2021).

While a plethora of parameters have been used in research, confusion remains around those providing the most favorable effect. To our knowledge, this is the first scoping review to collate, summarize and report on the different nGVS parameters used to improve postural control. Consensus on the optimum parameters has been identified as a critical next step to further this research (Herssens and McCrum, 2019; Haxby et al., 2020; Stefani et al., 2020; Lajoie et al., 2021; Pires et al., 2022). In this review, we look at the current evidence around parameter choice and give recommendations on future reporting to add rigor to nGVS research and facilitate meaningful comparison between studies.

## Method

The present scoping review was undertaken using the Arksey and O’Malley (2005) approach. A literature search was undertaken using: EBSCO (CINAHL plus, SPORTDiscus), Scopus, Ovid (AMED) and PubMed (Medline). We used the search terms (nGVS OR noisy galvanic stimulation OR Noisy vestibular stimulation OR galvanic vestibular stimulation OR GVS OR SVS OR stochastic vestibular stimulation) AND (gait OR walk* OR ambulation OR locomotion OR mobility OR balance OR stability OR posture*). Additional references were found by hand searching the reference lists of key articles. Studies were restricted to peer reviewed journals with full text available in English; no limit was placed on the publication date or study design.

Search results were imported into Covidence systematic review software (Veritas Health Innovation, Melbourne, Australia. Available at www.covidence.org). Articles were screened by title and abstract. The full text publication was reviewed independently by two reviewers (RM and DT) and eligibility determined according to the criteria in Table 1. If there was any uncertainty about inclusion, a third reviewer (PS) was consulted until a consensus was reached. The literature search was last performed on the 22/01/23.

**Table 1:** Scoping review inclusion and exclusion criteria.

	Inclusion criteria	Exclusion criteria
Population	Human	Animal studies
	Adults aged over 18 years	
Intervention	Bipolar noisy galvanic current applied bilaterally over mastoid processes	nGVS parameters set at a level with a goal of perturbing balance or gait
Control	No criteria	
Outcomes	Physiological gait or balance measures	
Trial design	Original primary data	Review articles
	Pre/post experimental design, crossover study, randomized controlled trial	Expert opinions
Data	Full text available	
	Peer reviewed journal		
	English		

Data were extracted directly from the text and tables into a customized template generated on Covidence software. Data extracted included: author, year of publication, study design, population, and the following nGVS parameters: skin preparation, conductive medium at the electrode skin interface, impedance restriction, electrodes used (material, size, surface area), electrode placement, waveform, frequency band, amplitude, method of optimization, duration of the stimulation and time period of assessment. Data were charted and grouped to synthesize key information regarding parameters, and a summary of the results was generated.

## Results

**Figure 2:**
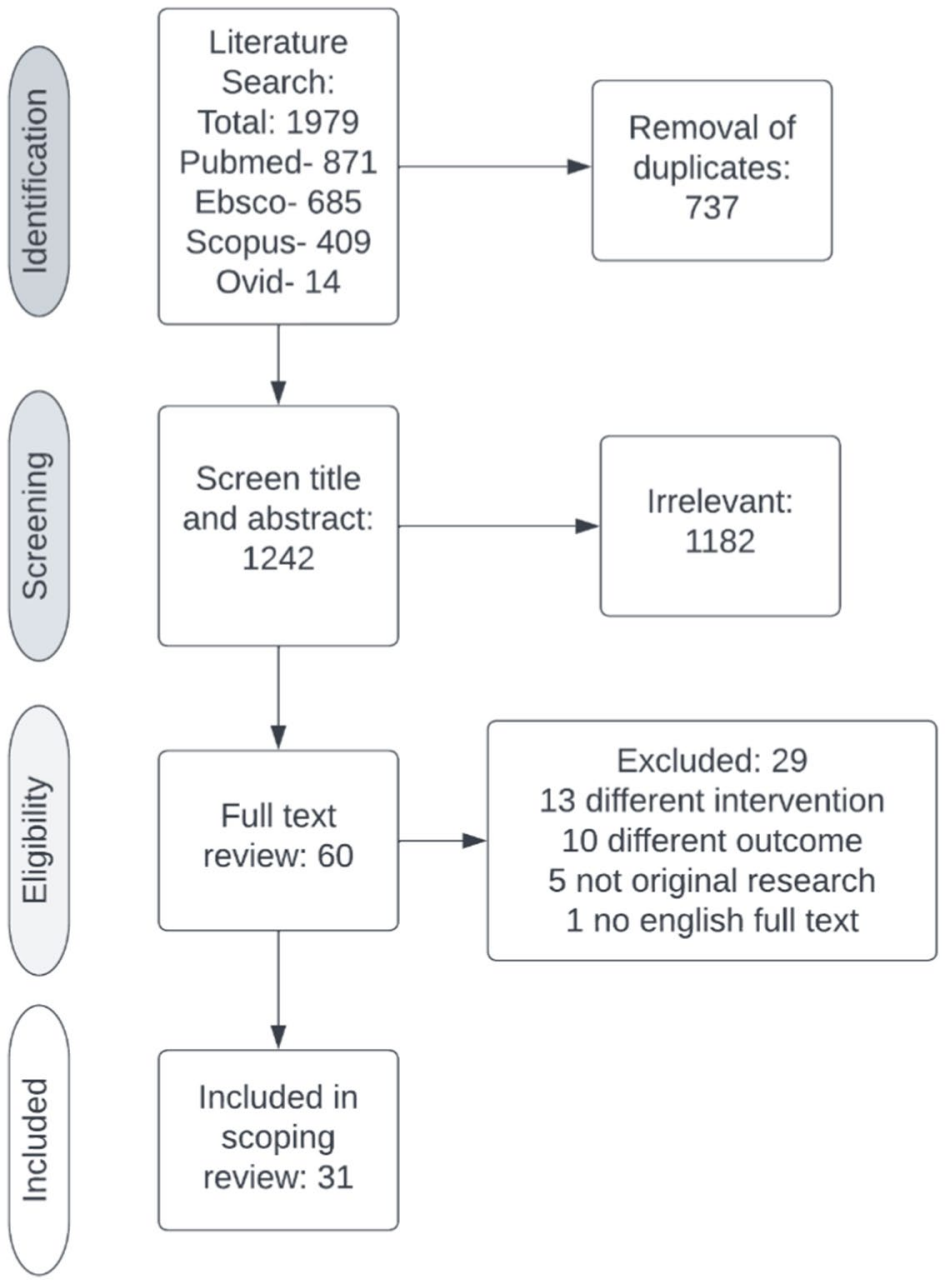
PRISMA flowchart.

After removal of duplicates, the electronic search yielded 1,242 citations. The title and abstract were screened against the inclusion and exclusion criteria (Table 1), excluding 1,182 studies. Two reviewers (RM, DT) completed a full text review on 60 studies. Twenty-nine studies were excluded, leaving 31 studies for data extraction (Figure 2). Table 2 contains a summary of the studies included in this review. Review findings are reported and discussed in an integrated analysis and discussion.

**Table 2:** A summary of the articles included in the parameter scoping review.

Study ID	Study design	Population description	*n*	Aim of study	Summary of findings
Asslander et al. (2021)	Randomized crossover	Healthy young	21	To investigate whether nGVS induced effects on body sway consistently follow a SR like bell shaped performance curve.	nGVS resulted in reduced postural sway in standing in most trials. However, the majority of participants did not demonstrate an SR pseudo bell shaped curve.
Chen et al. (2021)	Crossover	Healthy, BVP	16 healthy 10 BVP	To investigate the effects of nGVS in straight walking and 2 Hz head yaw walking in healthy and bilateral, vestibular hypofunction, in light and dark conditions.	nGVS reduced walking deviations in people with BVP. This was particularly evident in visually deprived conditions
Eder et al. (2022)	Double blind randomized controlled trial	BVP	23	To investigate potential synergistic effects of nGVS when combined with vestibular rehabilitation.	After 2 weeks vestibular rehabilitation training people with BVP demonstrated improved balance. There was no significant difference between those that received nGVS during the training and those that received sham treatment.
Fujimoto et al. (2016)	Block randomized pre/post experimental	Healthy older adults	30	To investigate the aftereffects of imperceptible nGVS on body balance in elderly adults.	During 3 h of nGVS there was reduced mean velocity at 1 h. Post nGVS there was a significant reduction in COP after 30 min that was maintained for 4 h. A further 30 min of nGVS added to the improvement and was maintained for a further 4 h.
Fujimoto et al. (2018)	Pre post experimental	BVP	13	To examine whether 30 min nGVS continues to improve balance after the cessation of stimulus in BVP patients	Reduced COP mean velocity was observed after 30 min nGVS. This effect was sustained for 3 h
Goel et al. (2015)	Randomized crossover	Healthy young adults	45	To investigate the effect of SVS on balance functions.	SVS at 46–53% of perceptual motion threshold of 1 Hz sinusoidal waveform, significantly improved balance in 70% of people (9/15, 13/15, 10/15). Responders demonstrated significantly improved balance in 4 different experimental studies.
Inukai et al. (2018a)	RCT	Healthy older adults	32	To assess the influence of nGVS on COP sway in community dwelling older adults during standing	Reduced sway path length, and mean sway velocity in nGVS group compared to control. Correlation between initial sway path length and balance measures during nGVS.
Inukai et al. (2018b)	Crossover	Healthy adults	18, 24, 16	To clarify the influence of nGVS on the center of pressure sway measurement in standing EO and identify the responders to nGVS	Subthreshold nGVS decreased sway path length during 30 s and 5 s stimulation in healthy young people. When divided into high and low initial sway path nGVS effect was only significant in those who had high initial sway path.
Inukai et al. (2020a)	RCT	Healthy adults	36	To elucidate the effects of nGVS on COP sway during one legged standing at different current amplitudes	200 μA nGVS reduced sway velocity and sway path length in healthy young people standing on one leg with EO. There was no significant effect for the 0 and 400 μA groups. Baseline values correlated with the stimulation effect in the 200 μA group only.
Inukai et al. (2020b)	Experimental	Healthy adults	24	To determine the effects of different floor surface and visual conditions on the stimulus effects of the nGVS intervention.	nGVS reduced AP sway velocity and sway path length at 0 min and 10 min after 6 x 30s standing EO on a firm surface but not with EC on foam.
Inukai et al. (2020c)	RCT	Healthy adults	26	To elucidate the after effect of nGVS on COP sway. To identify subjects for whom nGVS is effective.	Significant decrease in AP and ML COP velocity and sway path length compared to baseline during nGVS and at 10 min post nGVS. Significant correlation between baseline sway velocity and stimulation effect.
Iwasaki et al. (2014)	Crossover	Healthy adults, BVP	healthy 21 BVP 11	To examine the effect of imperceptible level of nGVS on postural performance in healthy subjects and patients with BVP	nGVS at an optimal amplitude reduced COP velocity, area, and RMS in healthy and BVP subjects. There was no significant difference in balance parameters at the non-optimal amplitude. 76% of healthy participants and 91% of BVP participants showed an optimal response to nGVS.
Iwasaki et al. (2018)	Experimental	Healthy, BVP	19 Healthy 12 BVP	To examine the effect of an imperceptible level of nGVS on dynamic locomotion in normal subjects as well as patients with BVP	nGVS had a significant effect, improving gait velocity, stride length and stride time in healthy and BVP as measured by a trunk worn sensor.
Ko et al. (2020)	Experimental	Healthy, BVP	10 Healthy 7 BVP	To investigate the effect of nGVS on posture and neural activity during standing and walking with 2 Hz head turning.	Healthy and BVP groups demonstrated a significant decrease in COP RMS with nGVS and head turning was significantly more coherent to 2 Hz with nGVS.
Lotfi et al. (2021)	Single blind RCT	Multiple Sclerosis	24	To compare the effectiveness of vestibular rehabilitation therapy or noisy galvanic stimulation for dizziness and balance responsiveness in MS.	The vestibular rehabilitation group demonstrated improved postural control. nGVS 30 min twice a week for 6 weeks did not improve balance. There was no significant difference between the nGVS group and the control group
Matsugi et al. (2020)	Sham controlled crossover	Healthy young adults	17	To investigate whether nGVS modulates body sway and muscle activity of the lower limbs depending on visual and somatosensory information from the foot using rubber-foam	1,000 μA nGVS increased body sway on foam. There was no significant difference on a firm surface, there was no significant difference with eyes open or closed.
Matsugi et al. (2022)	Single blind crossover	Healthy young adults	30	To investigate whether noisy galvanic vestibular stimulation (nGVS) modulates the vestibulo-ocular reflex (VOR) and whether this effect is correlated with the effect of nGVS on body sway.	VOR gain was significantly decreased at 200 μA, and sway path length significantly increased at 600 μA. No correlation observed between the effect of nGVS on COP and VOR related parameters.
Matsugi et al. (2022)	Double blind crossover	Healthy adults	17	To identify the changes in lower limb muscle activity and joint angular velocity during nGVS	nGVS altered the physical response in different standing postural conditions (exploratory study). During nGVS lower limb angular velocity was significantly decreased in the transverse direction when standing on a foam surface with EC
Mulavara et al. (2011)	Crossover	Healthy adults	15	To investigate the effect of two different frequencies of nGVS on postural sway	An optimal level of SVS was found in 8/15 people at 1-2 Hz and 10/15 at 0–30 Hz. There was no significant difference in balance performance between the two frequencies.
Mulavara et al. (2015)	Randomized crossover	Healthy adults	13	To investigate the effect of SVS on the postural response to perturbations during gait.	SVS at an optimal level reduced gait cycle time variability and increased trunk stability walking on an oscillating treadmill. Optimum peak current was at 35% of perceptual motion threshold
Nooristani et al. (2019a)	RCT	Healthy adults	36	To determine the effect of current density on postural control during nGVS.	nGVS significantly reduced sway path length and sway velocity immediately after stimulation with 3cm^2^ electrodes (high current density) but not with 35cm^2^ electrodes (low current density) or sham stimulation.
Nooristani et al. (2019b)	RCT	Healthy adults	28	To investigate the lasting effect of nGVS on postural stability.	Significantly reduced sway path and COM velocity in both nGVS and sham immediately after and 1 h after stimulation. No significant difference between groups.
Nooristani et al. (2021)	Randomized controlled trial	Older adults and older adults with vestibular impairment	24 Healthy older adults 12 Older adults with vestibular impairment	To determine the effect of nGVS on postural control in older adults with and without vestibular impairment. To examine the sustained effect of nGVS compared to sham stimulation	nGVS significantly reduced sway velocity and sway path length during and after stimulation compared to sham. There was a significantly greater effect in older adults with vestibular impairment compared to those with normal function.
Piccolo et al. (2020)	Crossover	Healthy adults	13	To determine whether SVS affects postural stability when balance is challenged during standing and walking tasks (EO). To compare the effect of amplitude optimization derived from sinusoidal or cutaneous threshold techniques.	10 s SVS demonstrated improved balance in balance challenged standing and reduced step width variability during gait. There was no significant effect between trials whether amplitude optimization was determined by motion perception (sinusoidal GVS) or cutaneous threshold.
Samoudi et al. (2015)	Double blind crossover	Parkinson’s disease	10	To investigate the safety and effects of SVS in patients with Parkinson’s disease.	Significantly reduced sway path standing with EC on foam and significantly reduced recovery time to perturbation with SVS compared to sham stimulation
Sprenger et al. (2020)	Crossover	BVP, healthy	30 BVP 24 healthy	To determine whether nGVS improves postural control in comparison to sham stimulus in context dependent conditions.	nGVS applied at 80% motion perception threshold did not significantly change standing balance on a firm surface with EO or EC or during a dual task. Standing on foam with EC nGVS resulted in significant deterioration in standing balance
Temple et al. (2018)	RCT (single blind)	Healthy adults	24	To determine whether SVS could improve short term adaptation to a locomotor task in a novel sensory discordant environment	7/12 participants responded to nGVS. nGVS resulted in faster times on a functional mobility test while wearing prism glasses and demonstrated faster adaption to a complex novel locomotion task with sensory discordance, compared to control group.
Woll et al. (2019)	Crossover	PPPV and Healthy	24 PPPV 23 Healthy	To investigate the effect of nGVS on patients with PPPV compared to healthy controls	The perceptual threshold for GVS was significantly reduced in PPPV compared to healthy controls. There was no significant difference in postural postural sway speed between nGVS and no nGVS conditions. Romberg quotient was significantly reduced with nGVS compared to the no GVS condition on both firm and foam surface conditions.
Wuehr et al. (2016a)	Crossover	BVP	13	To examine the effects of nGVS on dynamic walking stability in people with BVP	nGVS resulted in reduced gait variance and increased bilateral coordination, particularly during gait at slow speeds.
Wuehr et al. (2016b)	Crossover	Healthy adults	17	To examine the effects of nGVS on the walking performance in healthy adults walking with EC.	nGVS reduced gait variance and improved bilateral phase co-ordination during walking with EC at slow walking speeds only.
Wuehr et al. (2022)	Single blind crossover	Parkinson’s disease	15	To investigate the potential mode of action of the therapeutic effect of nGVS	At least half PD patients demonstrated reduced body sway compatible with SR.

AP, anterior–posterior; BVP, bilateral vestibulopathy; COM, center of mass; COP, center of pressure; EC, eyes closed; EO, eyes open; hrs, hours; nGVS, noisy galvanic vestibular stimulation; RMS, root mean squared; SR, stochastic resonance; SVS, stochastic vestibular stimulation; VOR, vestibulo-ocular reflex; PPPV, Persistent Perceptual Postural Vertigo.

## Integrated analysis and discussion

The parameters used in nGVS studies are described and how these may affect the efficacy of nGVS when used to treat people with postural control deficits, are evaluated.

### Electrodes and the electrode skin interface

#### Electrodes

Electrodes are the conductive surface delivering stimulation to the skin (Table 3). Manufactured from carbon rubber or silver/silver chloride, circular, oval or rectangular in shape, they have varied in size between a surface area of 0.5cm^2^ to 50cm^2^. The effect of electrode size during nGVS has been examined in only one study. Nooristani et al. (2019a) used identical stimulation parameters applied *via* an electrode of 35cm^2^ and an electrode of 3cm^2^. This generated the same stimulus, but different current densities at the skin-electrode interface. The smaller sized electrodes, providing higher current density over a smaller area, resulted in a significant improvement in balance compared to the 35 cm^2^ electrodes, that provided results that were no different to sham stimulation. Truong et al. (2023) modeled the electrical effect of both 3, and 35cm^2^ electrodes and reported the smaller electrode provided a more focal stimulation of the vestibular apparatus and temporal cortex, whereas there was more current loss *via* the skull and cerebrospinal fluid using a 35cm^2^ electrode. To afford further support for the observation that smaller electrodes provide more specific, localized stimulation of the vestibular system, the size, shape and material of electrodes, and details around the electrode-skin interface, should be clearly reported.

**Table 3:** Summary of electrodes used and the skin electrode interface.

Study ID	Electrode material	Electrode shape	Electrode size (cm^2^)	Skin prep	Conductive medium	Impedance (kOhms)
Iwasaki et al. (2014, 2018), Fujimoto et al. (2016, 2018), Woll et al. (2019)	NR	NR	NR	NR	NR	NR
Ko et al. (2020)	Carbon rubber	NR	NR	NR	Gel	NR
Sprenger et al. (2020)	NR	NR	0.5	NR	NR	NR
Matsugi et al. (2020, 2022)	Ag/ AgCl	NR	NR	NR	NR	NR
Wuehr et al. (2016a,b)	Conductive rubber	NR	NR	NR	Saline-soaked sponges	NR
Inukai et al. (2020a,b,c)	NR	Circular	1.75	NR	NR	NR
Nooristani et al. (2021)	NR	Circular	3	NR	Ten20 Paste	<10
Inukai et al. (2018a,b)	NR	Circular	3	NR	NR	NR
Nooristani et al. (2019a)	NR	Circular Rectangular	3, 35	NR	NR	NR
Mitsutake et al. (2022)	Ag/ AgCl	Square	9	NR	NR	<10
Asslander et al. (2021)	Rubber	NR	10	NR	Gel	NR
Lotfi et al. (2021)	Skintact Wet Gel Electrode	Circular	20	Cleaned (Nuprep)	Gel	<1
Eder et al. (2022)	Conductive rubber	Rectangular	24	NR	Saline-soaked sponges	NR
Samoudi et al. (2015)	NR	Oval	24	Cleaned (Nuprep)	Gel	<1
Wuehr et al. (2022)	Ag/ AgCl	Rectangular	24	Cleaned (abrasive gel)	NR	NR
Piccolo et al. (2020)	NR	Square	25	Cleaned and dried	Gel	NR
Chen et al. (2021)	NR	Rectangular	27.5	NR	NR	NR
Nooristani et al. (2019b)	NR	Rectangular	35	NR	Saline-soaked sponges	NR
Mulavara et al. (2015), Goel et al. (2015)	NR	Rectangular	50	Cleaned and dried	Gel	<1
Temple et al. (2018)	NR	Rectangular	50	Cleaned and dried	Gel	<1
Mulavara et al. (2011)	NR	Rectangular	50	Cleaned and dried	Gel	<0.6

NR, not reported.

#### The electrode skin interface

The connection between the skin and the electrode is affected by impedance. To deliver the desired current, impedance is kept at the lowest level possible, by skin preparation to reduce the resistance of dry skin cells and the stratum corneum, combined with the use of a conductive medium between the electrode and the skin (Table 3; McAdams et al., 1996; Abe and Nishizawa, 2021). At electrical frequencies lower than 1 Hz the impedance at the interface between the electrode and the electrolytic medium provides the greatest impedance to current, whereas at frequencies between 1 and 10,000 Hz the skin, and particularly the stratum corneum, produces the most impedance (McAdams et al., 1996). Fixed bandwidth nGVS contains frequencies within both these ranges (Table 4). While skin preparation and the skin/ electrode interface are paramount in electrical stimulation protocols, they are often unreported in the nGVS literature (Table 3). The conductive interface between the electrode and skin is either a saline soaked sponge (Wuehr et al., 2016a,b; Nooristani et al., 2019b; Eder et al., 2022) or electrode gel (Mulavara et al., 2011; Goel et al., 2015; Mulavara et al., 2015; Samoudi et al., 2015; Temple et al., 2018; Ko et al., 2020; Piccolo et al., 2020; Asslander et al., 2021; Lotfi et al., 2021). Most nGVS machines measure impedance and will signal to the user if it becomes too high. Higher impedance will either reduce the current that a machine is able to deliver to the vestibular system or, will draw a higher voltage to maintain the current amplitude (it is assumed that below the specified impedance threshold the relationship between frequency and impedance should not affect the current amplitude). Only seven studies mention the level and whether impedance is controlled: keeping impedance below 600 Ohms (Mulavara et al., 2011) or 1,000 Ohms (Goel et al., 2015; Mulavara et al., 2015; Samoudi et al., 2015; Temple et al., 2018) in five studies and below 10,000 Ohms in two (Nooristani et al., 2021; Mitsutake et al., 2022). Mulavara et al. (2011) provided a frequency analysis of their signal at the machine but to our knowledge no one has investigated the waveform frequency at the skin level to determine how the conductive interface at the skin influences the final stimulation output.

**Table 4:** Frequency band of nGVS.

Frequency band (Hz)	Study	Rationale for frequency band
Not reported	Matsugi et al. (2020, 2022)	Unreported
1–2	Mulavara et al. (2011)	Frequency of head motion during standing
0.02–10	Iwasaki et al. (2014, 2018), Fujimoto et al. (2016, 2018), Ko et al. (2020), Asslander et al. (2021), Chen et al. (2021)	Frequency of body sway in standing and covers the typical range of head motion signals
0.02–20	Sprenger et al. (2020), Woll et al. (2019)	Not reported
0–30	Eder et al. (2022), Mulavara et al. (2011, 2015), Goel et al. (2015), Piccolo et al. (2020), Wuehr et al. (2016a,b), Temple et al. (2018), Samoudi et al. (2015), Lotfi et al. (2021), Wuehr et al. (2022)	Proposed to stimulate vestibular hair cells and is the natural frequency bandwidth of the vestibular system.
0–250	Mitsutake et al. (2022)	Not reported
0–640	Nooristani et al. (2019a,b)	Increases excitability when applied to the cortex
0.1–640	Inukai et al. (2018a,b, 2020a,b,c), Nooristani et al. (2021)	Increases excitability when applied to the cortex

#### Summary of findings, electrodes, and electrode skin interface

Overall, details of the electrodes and electrode skin interface are not consistently reported. With the limited data available it appears that smaller electrodes arranged in a bipolar configuration may yield better postural control (Nooristani et al., 2019a). Basic science suggests that skin preparation and an interface medium to reduce skin impedance are a critical part of the stimulation process (McAdams et al., 1996; Abe and Nishizawa, 2021). As we move toward investigating the use of nGVS as either an orthotic to improve postural control or a means of enhancing rehabilitation, the features within the interface between the technology and the person are going to become more important (McLaren et al., 2016). In the people most likely to benefit from an nGVS intervention, the ear can already be a “busy” place, potentially housing hearing aids, glasses, mask loops and earrings. Small, unobtrusive, and tidy interfaces are likely to be more acceptable for regular or long-term use. This will need to be considered if this technology progresses to a consumer usability and commercial development stage.

### Waveform

**Figure 3:**
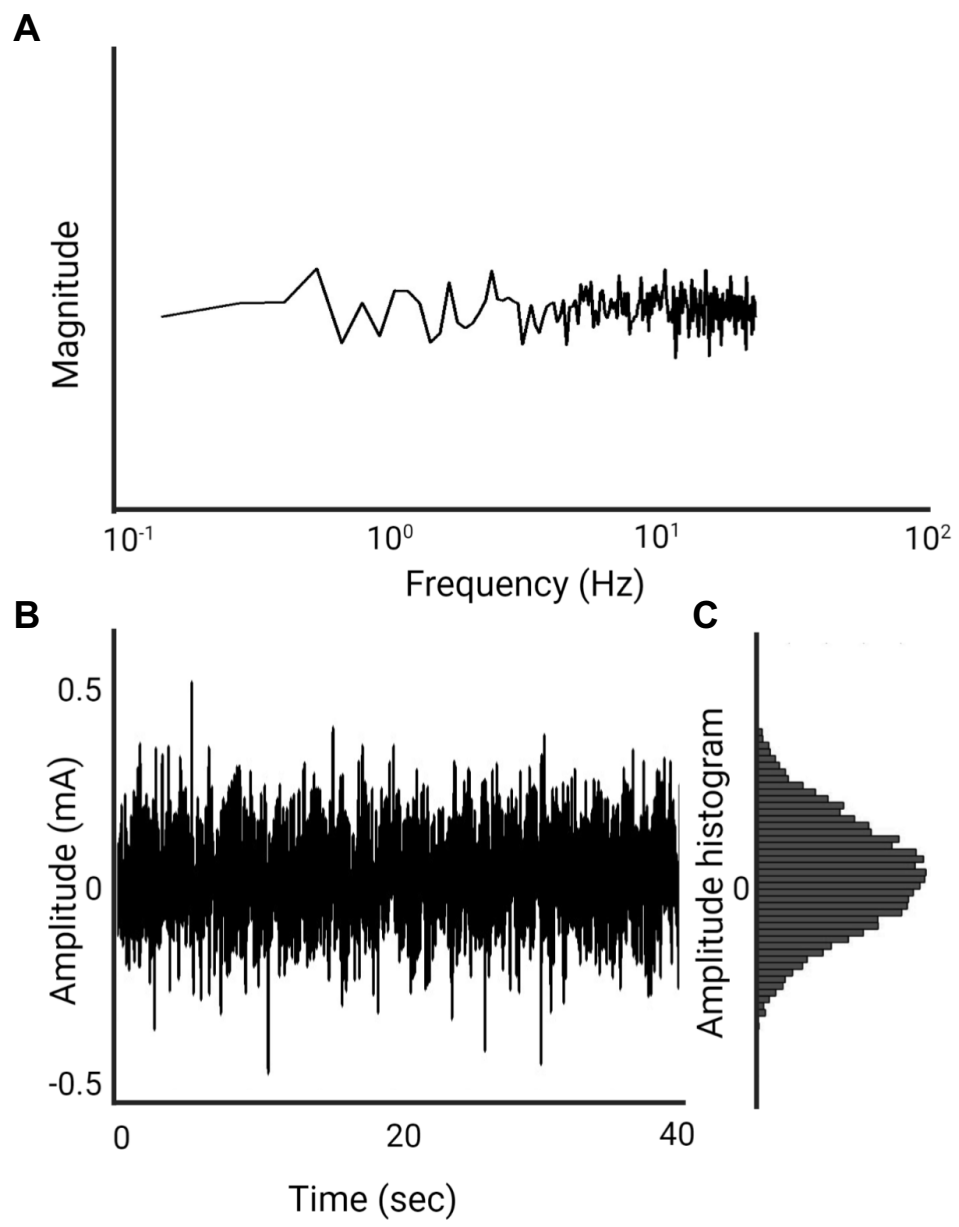
1 mA, 0–30 Hz Gaussian white noise nGVS signal (Created with BioRender.com). **(A)** Spectral frequency graph, the waveform contains frequencies between 0 and 30 Hz with equal intensity at each frequency. **(B)** Time plot of the signal. The amplitude of 1 mA means that 99% of all generated amplitude values are between 0.5 mA and −0.5 mA. **(C)** A histogram of the distribution of the signal amplitude. A random level of current generated for every sample is normally distributed, thus the probability density function follows a bell-shaped curve.

**Figure 4:**
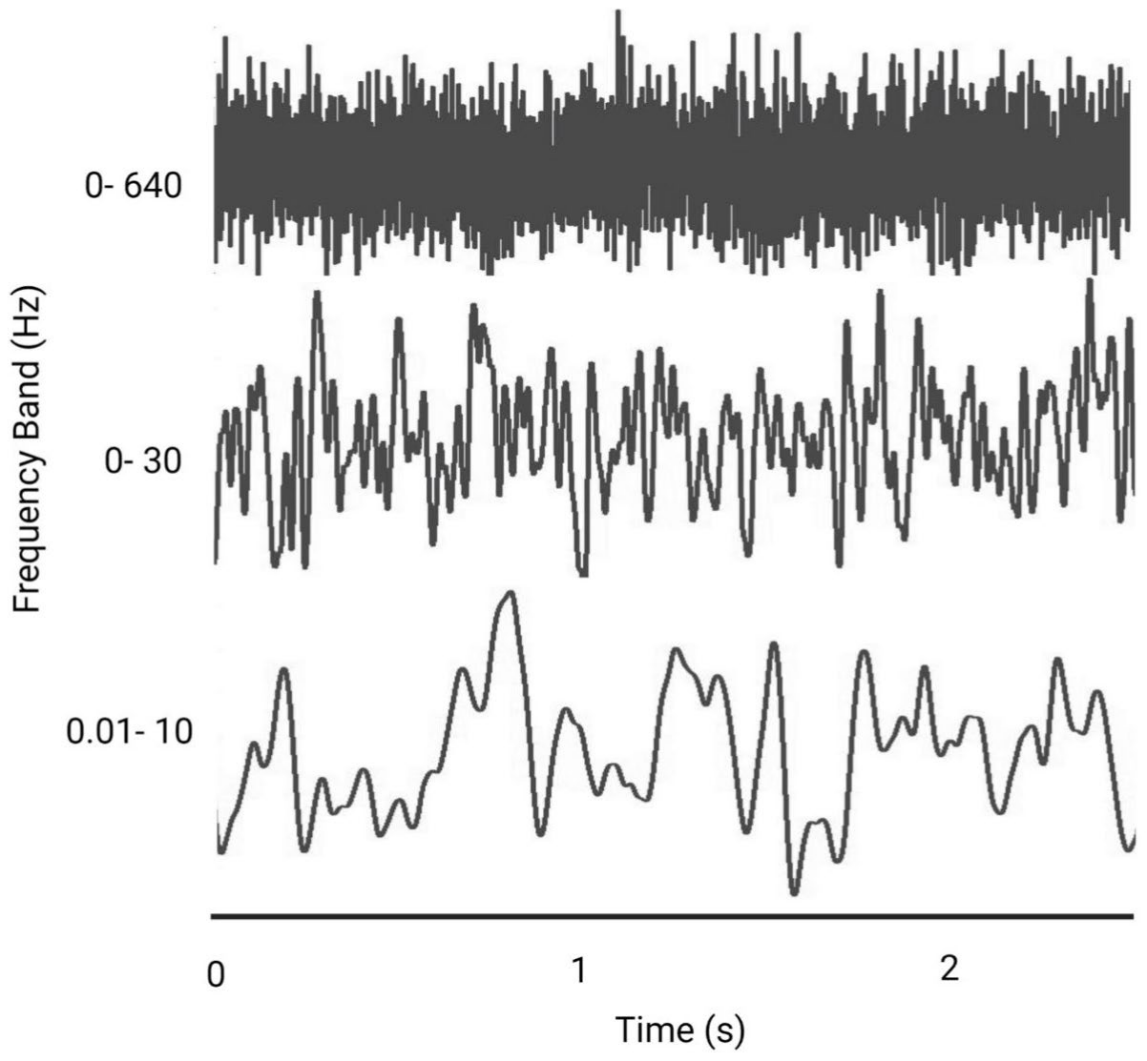
Representative figure comparing the effect of frequency bandwidth on a Gaussian white noise signal (Created with BioRender.com).

Noisy galvanic stimulation (nGVS) is a zero-mean noisy, alternating current delivered *via* electrodes over the bilateral mastoid processes. The zero-mean characteristic ensures that the stimulation does not introduce a constant bias or offset to the vestibular system (Stirzaker, 2005). nGVS and stochastic vestibular stimulation (SVS) are the most commonly used terms to describe this waveform in the literature. While there are numerous types of noise, the noise referred to in the nGVS literature is primarily white noise. White noise is a random signal with a flat power spectrum, meaning its power is distributed equally across all frequencies. This is similar to white light, which contains all visible wavelengths with the same energy (Marmarelis, 2012). The most common type of white noise referred to in relation to nGVS is Gaussian distribution (Figure 3). In the context of white noise, “Gaussian” refers to the amplitude distribution of the signal, which follows a Gaussian or normal distribution (Figure 3)(Marmarelis, 2012). Both the frequency distribution and distribution of current amplitude will influence the shape of the waveform (Figures 3, 4), and mathematically we can expect this to influence stochastic resonance (Hanggi et al., 1993). While not investigated in the postural control literature, Soma et al. (2003) found pink noise nGVS out- performed white noise when used to sensitize venous baroreceptors in response to postural change. Pink noise has also improved fine motor control in people with Parkinson’s disease (Kuatsjah et al., 2019). However, lack of clarity around the waveform used, imprecise use of nomenclature, and simultaneous variation of other parameters, make the influence of noise waveform on the efficacy of nGVS unclear.

#### Frequency bandwidth

nGVS current is delivered across a variety of frequency bandwidths, in which all frequencies within the bandwidth are represented. The benefits of nGVS for postural control have been seen over all these frequency bands, although it is possible that different frequency bands may be optimal for different people (Lee et al., 2021).

While the frequency band width has an influence on the shape of the noisy signal (Figure 4), to date there has not been a comprehensive comparison of the effect of different frequencies on performance (Stefani et al., 2020; Lajoie et al., 2021). Whether there is an optimum frequency band that will provide consistently improved results, and whether these frequency bands have different effects depending on the task (i.e., standing versus walking) is unknown (Wuehr et al., 2017; Inukai et al., 2018a; Lajoie et al., 2021).

Various rationales around head motion, physiology of the vestibular system or CNS function support the choice of different frequencies (Table 4). Specifically, the frequency of head motion during gait is reported as occurring around 0- 2 Hz (Hirasaki et al., 1999), whereas, the frequency of body sway is considered to sit at frequencies between 0.02-10 Hz (Asslander et al., 2021; Lajoie et al., 2021). A frequency band of 0- 30 Hz is the natural frequency of the vestibular system and is proposed to stimulate vestibular hair cells (Mulavara et al., 2011; Goel et al., 2015; Mulavara et al., 2015; Dlugaiczyk et al., 2019); and a frequency band of 0–640 Hz has been used to increase excitability when applied to the cortex (Terney et al., 2008).

In the only study comparing nGVS frequency bands, Mulavara et al. (2011) found that 8/15 participants improved their postural control with a 1–2 Hz bandwidth noise signal and 10/15 responded to a 0–30 Hz bandwidth white noise signal. There was no significant difference in the balance performance between the stimulation in the two frequency ranges. Stefani et al. (2020) found that the percentage improvement in balance was significantly larger for frequencies of 0–30 Hz compared to 0.1–640 Hz. However, the small number of studies, variation in stimulation parameters aside from frequency band, and inconsistency in outcome measures used, highlight the challenges of understanding optimum stimulation parameters.

#### Amplitude

An electric current is measured in amperes (A) and can be measured as peak amplitude (the highest value), peak-to-peak (the change from the highest to the lowest amplitude value) or as root mean squared (rms). A limitation of the literature is the failure in most cases to specify how amplitude has been measured, making it difficult to determine how comparable amplitudes are between studies.

nGVS to improve balance function has typically been delivered at a low or subthreshold amplitude; at an insufficient level to induce any cutaneous or vestibular (motion) afferent information. The amplitude must be set to a level that enhances the subthreshold neural signal without interfering with the ability of the network to detect suprathreshold signals (Collins et al., 1995). Regardless of whether nGVS works *via* the mechanism of stochastic resonance or direct stimulation of the vestibular afferents or hair cells, augmentation of the neural signal is only advantageous when it arrives at the appropriate rate at the precise time to enable accurate decoding in the central nervous system (Rieke, 1997). An amplitude that is too low will not facilitate a weak physiological signal, whereas an amplitude that is too high will provide too much stimulation to the vestibular system, making the signal nonsensical and risking the central nervous system degrading the weighting given to the afferent signal (Stacey and Durand, 2000).

Current amplitudes used in nGVS studies designed to improve postural control have varied from 100 to 1,200 mA (Table 5). Amplitude has been reported in the majority of studies and is generally considered a key variable in the optimization of the nGVS signal (Wuehr et al., 2018). A variety of methods have been employed to determine amplitude. While it is generally accepted that amplitude is important, the literature is divided on whether amplitude should be determined at a group level or individualized to each participant.

**Table 5:** Signal amplitudes and optimization methods used across studies.

Author	Optimization method	Amplitude (μA)
Nooristani et al. (2021)	None	200
Inukai et al. (2020a)	None	200, 400
Matsugi et al. (2022)	None	200, 600, 1,200
Inukai et al. (2020b)	None	400
Inukai et al. (2020c)	None	400
Inukai et al. (2018a)	None	400
Inukai et al. (2018b)	None	400, 1,000
Matsugi et al. (2020)	None	1,000
Nooristani et al. (2019a)	None	1,000
Nooristani et al. (2019b)	None	1,000
Eder et al. (2022)	Multiple amplitudes	330 +/− 203 (BVP)
Mulavara et al. (2015)	Multiple amplitudes	238 +/− 154
Goel et al. (2015)	Multiple amplitudes	100–500
Iwasaki et al. (2018)	Multiple amplitudes	341.1+/− 46.6725 +/− 79.9 (BVP)
Iwasaki et al. (2014)	Multiple amplitudes	281.2 +/− 39.8455.6 +/− 81.8 (BVP)
Fujimoto et al. (2018)	Multiple amplitudes	454 +/− 55 (BVP) 462 +/− 65 (BVP)
Fujimoto et al. (2016)	Multiple amplitudes	178.8 +/− 9.1
Wuehr et al. (2022)	Multiple amplitudes	300 (Parkinson’s Disease)
Mulavara et al. (2011)	Multiple amplitudes	260 +/− 18 (0–30 Hz) 212.5 +/−14 (1–2 Hz)
Ko et al. (2020)	Multiple amplitudes	Not reported
Chen et al. (2021)	Multiple amplitudes	Not reported
Asslander et al. (2021)	Multiple amplitudes	Not reported
Piccolo et al. (2020)	50% motion threshold	304 +/− 81
Temple et al. (2018)	50% motion threshold	Not reported
Sprenger et al. (2020)	80% motion threshold	384 (Healthy), 648 (BVP), 440 (BVP low threshold), 912 (BVP high threshold)
Woll et al. (2019)	80% motion threshold	376 +/−40 (Healthy), 248 +/−38 (PPPD)
Samoudi et al. (2015)	100% motion threshold	500 +/− 255
Piccolo et al. (2020)	80% cutaneous threshold	249 +/− 84
Wuehr et al. (2016a)	80% cutaneous threshold	381.5 +/− 38.3 (BVP)
Wuehr et al. (2016b)	80% cutaneous threshold	324.2 +/− 28.5
Mitsutake et al. (2022)	80% cutaneous threshold	370 +/− 60
Lotfi et al. (2021)	90% cutaneous threshold	540 +/− 400 (Multiple Sclerosis)

BVP, Bilateral vestibulopathy.

Using a set amplitude at a value that is most likely to be subthreshold for the group is the most straightforward method of deciding on the amplitude. Using this method, a machine can be pre-set and used with minimal training. The disadvantage of a set amplitude is that it may not be facilitatory for all. Ten studies set amplitude at a group level. One study used a 1,200 μA amplitude (Matsugi et al., 2022), four studies used a 1,000 μA amplitude (Inukai et al., 2018b; Nooristani et al., 2019a,b; Matsugi et al., 2020), one at 600 μA (Matsugi et al., 2022), five studies have used a set amplitude of 400 μA (Inukai et al., 2018a,b, 2020a,b,c), and three studies 200 μA (Inukai et al., 2020a; Nooristani et al., 2021; Matsugi et al., 2022). The varying nature of study designs and the sensory conditions utilized during assessment make it difficult to draw credible conclusions between studies (Matsugi et al., 2020; McLaren et al., 2022). Of the current amplitudes set at a group level, 200 μA demonstrated significantly reduced postural sway in two studies (Inukai et al., 2020a; Nooristani et al., 2021), and no change in a further study (Matsugi et al., 2022); 400 μA provided noted improvements in balance in four studies (Inukai et al., 2018a,b, 2020b,c) with no effect in one study. At 1,000 and 1,200 μA, the results were variable; balance improved in one study (Inukai et al., 2018b), deteriorated in one study (Matsugi et al., 2020) but had no effect (Nooristani et al., 2019b; Matsugi et al., 2022), or had limited effect (Nooristani et al., 2019a) in three other studies.

In their ground-breaking primate work investigating individual sensory thresholds to sinusoidal galvanic vestibular stimulation, Kwan et al. (2019) recorded stimulation thresholds between 400 and 600 μA. Although there are limitations in applying the findings of sinusoidal galvanic vestibular stimulation to nGVS, this highlights uncertainty about whether set amplitudes used in some nGVS studies may be suprathreshold and are thus unlikely to induce stochastic resonance. In addition, while using a set threshold for all participants is appealing it may not be sensitive enough to provide optimisation of the signal to gain the most favorable result for individual participants.

In keeping with the theory of stochastic resonance, other studies have used various methods to individualize the amplitude to participants. This has been done by testing an array of amplitudes and choosing the amplitude that improves standing sway measures the most (Mulavara et al., 2011; Iwasaki et al., 2014, 2018; Mulavara et al., 2015; Fujimoto et al., 2016, 2018; Asslander et al., 2021; Chen et al., 2021), or by calculating amplitude at a percentage of the cutaneous sensory threshold (Wuehr et al., 2016a,b; Piccolo et al., 2020; Lotfi et al., 2021; Mitsutake et al., 2022) or a percentage of the vestibular (motion) threshold of 1 Hz sinusoidal GVS stimulation, to determine the optimum signal (Goel et al., 2015; Mulavara et al., 2015; Samoudi et al., 2015; Temple et al., 2018; Piccolo et al., 2020; Sprenger et al., 2020).

The underlying pathology also appears to influence the current amplitude that is optimal for individuals. Studies using individualized optimization have had a mean amplitude of 397 +/−167 μA, which is close to the value most commonly used in studies that set the amplitude at a group level. Optimized studies involving healthy subjects used a mean current amplitude of 309 +/− 83 μA whereas the mean amplitude for people with bilateral vestibulopathy (BVP) was 494 +/− 131 μA. A higher mean amplitude for people with an underlying vestibular diagnosis suggests that the integrity of the vestibular system should be considered when planning stimulation of clinical populations, to give the greatest chance of success.

Examining the means of individualizing the amplitude using a wide range of amplitudes and testing the sway parameters of each amplitude to find the optimum amplitude, could be the most specific method of determining optimal amplitude and has been the gold standard against which other methods have been measured (Iwasaki et al., 2014, 2018; Goel et al., 2015; Mulavara et al., 2015). However, it is time consuming and requires a laboratory-based setting to determine the optimum parameters, meaning it cannot be translated easily into the clinical setting. Fujimoto et al. (2018) used this technique in sessions 2 weeks apart. Of 13 participants, only 3 participants had the same optimum amplitude in both sessions, suggesting there are factors involved in the application of the stimulation or neural excitability that affect response to stimulation on a day-to-day basis.

Five studies have used vestibular motion perception to set amplitude. The theory behind this method of optimization is that it tests the excitability and integrity of the vestibular nerve and sets the current amplitude accordingly. A 1 Hz sinusoidal GVS waveform is delivered and the amplitude at which an individual senses mediolateral motion, or is observed moving on a force plate, is taken as the sensory threshold. A percentage of this threshold amplitude (between 50% and 100%) is then applied to a nGVS waveform. This could be done in a clinical setting as the perception of movement has been found to be as sensitive as measuring movement of the center of mass on a forceplate (Goel et al., 2015). However, a disadvantage is that this method requires a machine that can deliver both a GVS and nGVS waveform, and the risk of falls - particularly in those with impaired balance already (BVP and older adults). Studies using a 100% motion perception threshold have resulted in a deterioration in balance (Pavlik et al., 1999) or had no effect on postural control, and led to nausea in people with Parkinson’s disease who were medicated (Samoudi et al., 2015). nGVS at 80% motion sensation perception had no effect when standing on a firm surface with eyes open or closed (Woll et al., 2019; Sprenger et al., 2020) and led to increased sway standing on a foam surface (Sprenger et al., 2020). However, studies using an amplitude of 50% of motion perception were effective at improving postural control (Temple et al., 2018; Piccolo et al., 2020). Mulavara et al. (2015) investigated balance at a range of nGVS amplitudes and compared their results with the motion perception threshold, finding no significant correlation (*p* > 0.05) between optimal nGVS stimulation and perceptual threshold amplitude. In their study the average peak current amplitude that resulted in improved balance performance was 35% of the motion perception threshold. By contrast, Goel et al. (2015) found the optimal amplitude determined by exposure to multiple current amplitudes to be equivalent to 50% of the motion perception threshold. While conceptually sound as a method that investigates the responsiveness of the vestibular nerve to stimulation and sets the stimulation current amplitude accordingly, results have been inconsistent and further research is needed to establish the adequacy of this method of individualization.

The cutaneous threshold, or the point at which nGVS starts to cause cutaneous sensation under the electrodes, has also been used as a method of optimization (Wuehr et al., 2016a,b; Lotfi et al., 2021; Mitsutake et al., 2022). The advantage of using cutaneous sensory threshold to determine the optimum amplitude is that it can be easily done outside a laboratory setting, it only requires one waveform and there is a low risk of delivering stimulation at a level that may cause unexpected imbalance. At 80% of the cutaneous threshold, studies have demonstrated improvements in gait stability in both older healthy adults and those with BVP. Studies using balance measures at a range of amplitudes to find the optimum that have also tested the sensory threshold, support this finding. Iwasaki et al. (2014, 2018), found the optimum amplitude to be 83 and 85% of the sensory threshold, respectively. While this method has the benefit of providing a quick and easy method of optimization, the relationship between cutaneous sensation and vestibular function has not been established and the theoretical underpinnings of this method require validation.

#### Summary of the nGVS waveform used to enhance postural control

Amplitudes of nGVS between 100 and 1,200 μA have been used to improve postural control, with the most beneficial amplitudes appearing to sit in the lower end of this range. Individualisation of the stimulus amplitude appears to be important, particularly in participants with impaired vestibular function who may require a stronger stimulus intensity to receive the benefits of nGVS (Iwasaki et al., 2014, 2018; Sprenger et al., 2020). There is no one single method of tuning the signal amplitude to the individual that is obviously superior at this point.

A variety of frequency bands have been used to create the noisy signal, to date there has been insufficient systematic research investigating nGVS frequency bands to determine if there is an optimum frequency band that produces superior postural control (Lee et al., 2021). The solitary review on this topic determined that, a narrower bandwidth nGVS (0-30 Hz), demonstrated significantly greater postural control performance when compared to a wider bandwidth (0.1–640 Hz) (Stefani et al., 2020). Uniformity of terminology and provision of frequency and amplitude characteristics of the stochastic signals used, when reporting on nGVS will aid future inquiry into the effect of the waveforms.

### Duration and timing of stimulation

Most studies have looked at the coincident effect of nGVS and its effect on balance while actively stimulating the vestibular system (Table 6). The consensus from these studies is that nGVS can immediately reduce sway in standing (Pal et al., 2009; Mulavara et al., 2011; Iwasaki et al., 2014, 2018; Goel et al., 2015; Samoudi et al., 2015; Fujimoto et al., 2018; Inukai et al., 2018a,b; Nooristani et al., 2019a,b; Inukai et al., 2020a,b; Ko et al., 2020; Piccolo et al., 2020; Asslander et al., 2021) and improve gait parameters (Mulavara et al., 2015; Wuehr et al., 2016a,b; Iwasaki et al., 2018; Temple et al., 2018; Ko et al., 2020; Piccolo et al., 2020; Chen et al., 2021). These findings support the potential for nGVS to be used as an orthotic to support balance.

**Table 6:** Duration and timing of stimulation.

Study ID	Duration	Measured
Iwasaki et al. (2018)	Not reported	Baseline; During
Temple et al. (2018)	Not reported	Baseline; During
Piccolo et al. (2020)	Not reported	Baseline; During
Inukai et al. (2018b)	5 s, 30 s	Baseline; During; Straight after
Chen et al. (2021)	10 s	Baseline; During
Sprenger et al. (2020)	20 s	Baseline; During
Woll et al. (2019)	20 s	Baseline; During
Goel et al. (2015)	22 s	Baseline; During
Mulavara et al. (2011)	22.5 s	Baseline; During
Mulavara et al. (2015)	25 s	Baseline; During
Iwasaki et al. (2014)	30 s	Baseline; During
Inukai et al. (2018a)	30 s	Baseline; During
Inukai et al. (2020a)	30 s	Baseline; During
Inukai et al. (2020c)	30 s	Baseline; During
Wuehr et al. (2022)	30 s	Baseline; During
Mitsutake et al. (2022)	30 s	Baseline; During
Asslander et al. (2021)	30 s and 60 s	Baseline; During
Inukai et al. (2020b)	6× 30 s	Baseline; Straight after; Minutes later
Matsugi et al. (2020)	40 s	Baseline; During
Matsugi et al. (2022)	50 s and 70 s	Baseline; During
Wuehr et al. (2016a)	2 min	Baseline; During
Wuehr et al. (2016b)	2 min	Baseline; During
Ko et al. (2020)	6 min	Baseline: During
Fujimoto et al. (2018)	30 min	Baseline; Straight after; Hours later
Nooristani et al. (2019a)	30 min	Baseline; Straight after
Nooristani et al. (2019b)	30 min	Baseline; Straight after; Hours later
Nooristani et al. (2021)	30 min	Baseline; During; Straight after
Fujimoto et al. (2016)	2× 30 min or 3 h	Baseline; During; Straight after; Hours later
Lotfi et al. (2021)	30 min twice a week for 6 weeks	Baseline; After a sleep
Eder et al. (2022)	30 min three times a week for 2 weeks	Baseline; After a sleep
Samoudi et al. (2015)	<3 h	Baseline; During

A limitation in studies to date is the short duration of application - seconds to minutes. Only one study has investigated the effect of long duration nGVS over the stimulation period. Fujimoto et al. (2016), applied nGVS for 3 h and assessed balance at 1 and 2 h during the application. While there was a significant reduction in sway velocity after 1 h of nGVS, after 2 h this change was no longer significant. This is an important area for further investigation to determine whether the ameliorating effects of nGVS continue during prolonged application.

Residual effects on standing balance immediately after nGVS stimulation demonstrate a continued positive effect on standing balance and sway velocity (Fujimoto et al., 2016, 2018; Inukai et al., 2020c; Nooristani et al., 2021). Significant enhancement of balance has been observed 10 min after stimulation (Inukai et al., 2020b,c) and 30-min post-stimulation (Fujimoto et al., 2016, 2018), with improvements continuing to be significant for 3 hours after a 30-min period of stimulation (Fujimoto et al., 2016, 2018).

Two studies have used nGVS over a period of weeks (Lotfi et al., 2021; Eder et al., 2022). While neither of these studies have found a significant change in postural control with nGVS compared to their control groups, both studies contribute to our understanding of how we could make repeated application of nGVS more effective. Lotfi et al. (2021), investigated 30 min nGVS twice a week for 6 weeks in people with Multiple Sclerosis. They compared the treatment to vestibular rehabilitation and a no treatment group. They found vestibular rehabilitation significantly improved balance and there was no significant difference between receiving nGVS and no treatment. However, the subthreshold nGVS treatment was given while individuals lay supine. As the vestibular apparatus is relatively inactive in this stationary well supported position, it is possible that there was little vestibular afferent activity for subthreshold stimulation to augment in this treatment protocol. This pattern has also been seen in an MRI study. Helmchen et al. (2019) found no evidence of a change in cortical activity when participants received nGVS while lying supine with the head immobilized within an MRI machine. This supports the theoretical basis of stochastic resonance as the means behind nGVS, as without head movement there is little vestibular afferent signal to facilitate.

In comparison, Eder et al. (2022) investigated whether nGVS applied during vestibular rehabilitation over a 2-week period produced a synergistic effect compared to standard vestibular rehabilitation, in a randomized controlled pilot study. While nGVS augmentation of treatment was feasible, they found no difference between groups. Notably, while there was a reduction in base of support in both groups during closed eye walking, neither group demonstrated a significant change in postural sway measurements standing on foam with eyes closed. It is possible that this test is too challenging for people with bilateral vestibulopathy (Iwasaki et al., 2014; Sprenger et al., 2020; McLaren et al., 2022). Alternatively, this may indicate a difference in the way the CNS processes vestibular information in standing and walking or a targeted effect of vestibular rehabilitation toward mobility.

#### Summary of duration and timing used to enhance postural control

Short duration coincident nGVS appears to improve postural control in standing (Mulavara et al., 2011; Goel et al., 2015; Inukai et al., 2018a,b; Iwasaki et al., 2018; Inukai et al., 2020a,b,c; Asslander et al., 2021) and walking (Mulavara et al., 2015; Wuehr et al., 2016a,b; Temple et al., 2018; Piccolo et al., 2020; Chen et al., 2021), with significant effects lasting for hours after stimulation (Fujimoto et al., 2016, 2018). Intermittent nGVS over a period of weeks appears to be a safe treatment that is well tolerated (Lotfi et al., 2021; Eder et al., 2022). The efficacy of longer periods of nGVS is yet to be established.

### Limitations

This scoping review was limited to studies that have used a postural control measure that will be more responsive to the vestibular influence on the vestibulospinal reflexes (VSRs) and *via* the vestibulospinal tract. However, the vestibular system has a direct impact on postural control *via* both the VSRs and the vestibulo-ocular reflexes (VORs). While some studies have found the greatest benefit of nGVS was demonstrated with the eyes closed, eliminating the influence of the VOR on balance (McLaren et al., 2022), and others have found no effect of the visual condition (Matsugi et al., 2020), in most real-life situations the stabilizing role of the VOR for vision plays a critical role in balance and postural control. However, to date we do not have evidence that a change in postural control with nGVS is associated with a the change in the VOR (Matsugi et al., 2022).

Examining the effect of nGVS on vestibulo-ocular control can help us understand how nGVS influences the vestibular apparatus. As with the effect of nGVS on postural control, the effects on the VOR have also been contradictory. Some studies investigating the effects on otolith-ocular responses have demonstrated positive results. Iwasaki et al. (2017) found that nGVS significantly increased the amplitude of the ocular vestibular evoked myogenic potential (oVEMP) responses in 79% of ears. Serrador et al. (2018) found that imperceptible nGVS resulted in a significant increase in ocular counter roll gain in older adults, and Keywan et al. (2018) demonstrated a reduced vestibular perceptual threshold in the roll plane, and translational movement (Keywan et al., 2019). These studies indicate more sensitive vestibular function during active nGVS (Keywan et al., 2020), and support the theory that nGVS preferentially stimulates the irregularly firing afferents originating at the otolith organs (Keywan et al., 2019).

In contrast Matsugi et al. (2022) found that nGVS significantly reduced rather than increased the video head impulse test (vHIT) gain to horizontal head impulses, indicating suppression of the VOR with nGVS. The reasons for these contradictory results are not clear. As the frequency band of the nGVS stimulus in Matsugi et al. (2022) was not disclosed, and they used set amplitudes, there could be parameter based differences. Also, in both Matsugi et al. (2022) and Serrador et al. (2018), young healthy subjects demonstrated no change in ocular counter roll response to nGVS (Serrador et al., 2018) or vHIT (Matsugi et al., 2022). The young and healthy may demonstrate a ceiling effect and have little capacity for improvements in oculomotor control. This could also be an indication that the physiological response to nGVS is specific to the particular parts of the vestibular anatomy. The oVEMP and ocular counter roll following the initial head movement—both of which have shown a positive response to nGVS—primarily test the function of the utricle, whereas the horizontal vHIT is a test of the horizontal semicircular canals. Research looking at the effect of nGVS on ocular control provides helpful insight into the theoretical basis for this modality but needs context to be understood in relation to the influence on postural control. Future work looking at the role of nGVS-facilitated gaze stabilization and the influence this has on balance will help clarify the role of the vestibular system in postural and oculomotor control, and the physiological effects of nGVS on different anatomical sites of the vestibular system.

Task specificity during tuning of the nGVS signal has not been clearly addressed in the literature (Herssens and McCrum, 2019). To date, the optimization of nGVS for postural control has primarily been done in standing (Iwasaki et al., 2014; Goel et al., 2015; Mulavara et al., 2015; Chen et al., 2021) with only one study using gait velocity to optimize the nGVS amplitude (Iwasaki et al., 2018). In two comparable studies, Iwasaki et al. (2014, 2018) used the same 0.02–10 Hz frequency band tested across multiple amplitudes to optimize the stimulus amplitude. Iwasaki et al. (2014) used standing sway to determine the optimal amplitude and found that the mean optimum amplitude was 281 +/−40 μA in a healthy population and 456 +/− 82 μA in people with BVP. In a subsequent study Iwasaki et al. (2018) used the amplitude that improved gait velocity the most, to determine the optimum amplitude and found the mean optimum amplitude was much higher, 341 +/− 46.6 μA in a healthy population and 725 +/− 79.9 μA in people with BVP. This comparison suggests that the task may influence the choice of parameters, a gap in the literature that will be important to address as we move toward clinical trials of nGVS.

It is becoming evident that nGVS is unlikely to be a treatment suitable for improving postural control in every individual. Inukai et al. (2018a, 2020a), concluded that subjects who have a longer sway path at baseline appear to show a larger stimulation effect and Nooristani et al. (2021) found that nGVS induced significantly greater improvement in sway velocity in older adults with vestibular impairment (who would be assumed to have poorer balance), compared to older adults with normal vestibular function. Supporting this theory, in studies of healthy people, Fujimoto et al. (2019), Temple et al. (2018), and Goel et al. (2015) found only approximately 2/3 of healthy adults demonstrated an optimal response to nGVS. However, 45/46 participants with bilateral vestibulopathy demonstrated an improvement in stability when nGVS was applied (Iwasaki et al., 2014, 2018; Wuehr et al., 2016a,b; Fujimoto et al., 2018; Ko et al., 2020, Chen et al., 2021). This is hypothesized to be due to a ceiling effect, whereby nGVS is more effective in those with sub-optimal vestibular input such as vestibular disorders or older adults who are more likely to have age related presbyvestibulopathy (Inukai et al., 2018a, Nooristani et al., 2021). Or alternatively, non-responders may have an inherently low weighting for vestibular sensory contributions (Goel et al., 2015). Conversely, Schniepp et al. (2018) found that nGVS lowered the VSR threshold in individuals with vestibulopathy with residual function but no response to nGVS in those with complete loss of vestibular function bilaterally, indicating that some residual vestibular function is required for a response to nGVS. These findings highlight the importance of targeting nGVS to the populations that are most likely to benefit from this intervention, particularly those with poor balance or impaired vestibular function but without complete vestibular loss.

Overall, nGVS appears promising as a technology to facilitate vestibular function and improve gait and balance in people with balance deficits (Lajoie et al., 2021). However, a limitation of research to date is not only the vast array of different parameters that have been used, and a lack of consensus on optimum stimulus parameters, but also the complexities of the vestibular contribution to stability. CNS weighting of afferent inputs in the balance response, individual variances in responsiveness and the effects of the task all influence the benefits gained from nGVS (Herssens and McCrum, 2019).

### Recommendations

As a promising adjunct to balance rehabilitation, the progress of nGVS as a therapeutic tool is limited while the optimal parameters are not understood. There is currently insufficient evidence to develop a clinical guideline (AGREE Next Steps Consortium, 2017). However, looking to the future, researchers can bring us closer to that position by ensuring complete and transparent reporting of the parameters likely to influence the stimulation effect (Table 7) as we work toward developing consensus on optimum stimulus parameters.

**Table 7:** Key nGVS parameters for reporting.

Parameter	Variables to report	Intent	Uncertainties/ Next steps
Skin preparation	Cleaning, abrasion, hair removal, products used	Skin preparation and conductive medium affect impedance	Determine a maximum acceptable impedance level and consistently report in studies
Electrodes	Size, shape, material	Electrode size, shape and human interface may influence the specificity of the electrical stimulation and current density influencing stimulation effect	Further confirmatory studies looking at the effect of electrode site specificity and current density on electrical stimulation. Reporting electrode size and shape to enable meta- analysis in the future
Conductive medium between electrodes and skin	Conductive medium, products used. Impedance level	Conductive medium and skin contact affect impedance	Tidy and efficient conductive mediums will play a large role in the acceptability of nGVS as a neuroprosthetic or adjunct to rehabilitation
Waveform	Hardware (make and model), noise features (frequency band, distribution), description of how the waveform is produced if not a programmable commercial machine function.	Delivered waveform may not be equivalent to that of the machine settings, depending on how the waveform is manufactured and filtered.	Assess efficacy of different frequency bands and noise distribution.
Frequency	Bandwidth, and distribution	Frequency band is consistently reported in the literature. However, there is currently insufficient literature to determine whether frequency bandwidth influences postural control.	Specifically test the effect of nGVS frequency bandwidth on balance and gait parameters
Amplitude	Amplitude and method of calculating (i.e., peak, peak to peak, RMS).	Clearly reporting the method used to determine amplitude will enable us to compare studies more easily and determine optimum parameters for different populations. Reporting on the cutaneous threshold/ and motion perception threshold to 1 Hz GVS will help us whether these are valid methods of optimization.	Subthreshold stimulation appears to be the preferential means of enhancing balance and gait stability. Clinical populations such as those with BVP appear to have higher stimulation thresholds. This requires further investigation.
Method of optimization (if used)	Lower and upper bounds of assessment when using stepwise methods. Cutaneous threshold and vestibular motion threshold (GVS). Task used in optimization and criteria used to determine task specific optimization.	Provide further understanding of the role of optimization and enable comparison between populations and studies.	Investigate whether optimization is task specific (i.e., standing vs. walking). Develop methods of optimization utilizing equipment readily available in clinical environments.
Duration of stimulus	Duration in hours/min/s, washout period between trials	Examine effect of nGVS over longer periods of application	Understand the effect of nGVS as an orthosis and as an adjunct to rehabilitation as well as the sustained effects of this treatment.

## Conclusion

While nGVS parameters appear important, the ability to draw robust conclusions about the selection of optimal parameters is hindered by limited systematic evaluation of parameter settings within and across experiments; and variability in individuals’ response to nGVS. Understanding the optimum parameters to stimulate balance and gait responses will enable us to investigate the use of nGVS as a clinical tool to augment balance rehabilitation. However, before we can use nGVS as a therapeutic tool we need to sort out the science that will influence its clinical application. In response to the lack of research into the optimum parameters of nGVS, we propose a guideline (Table 7) for the accurate reporting of nGVS parameters, as a first step toward establishing standardized stimulation protocols.

## Author contributions

RM and DT conceptualized the study. RM performed the literature search. RM and DT independently reviewed the articles. RM extracted and critically reviewed the data and wrote the first draft of the manuscript. IN provided his expertise and critical feedback in the signal processing elements of the paper. PS, RT, and DT provided feedback at all stages of manuscript development. All authors contributed to the article and approved the submitted version.

## Funding

This work was supported by the Health Research Council of New Zealand grant numbers, HRC22/363 and HRC19/632.

## Conflict of interest

The authors declare that the research was conducted in the absence of any commercial or financial relationships that could be construed as a potential conflict of interest.

## Publisher’s note

All claims expressed in this article are solely those of the authors and do not necessarily represent those of their affiliated organizations, or those of the publisher, the editors and the reviewers. Any product that may be evaluated in this article, or claim that may be made by its manufacturer, is not guaranteed or endorsed by the publisher.
